# Explaining Retinal Susceptibility to Diabetes Through Photoreceptor Biology

**DOI:** 10.3390/ijms27094065

**Published:** 2026-05-01

**Authors:** William C. Carter, Rithwick Rajagopal

**Affiliations:** John F. Hardesty, MD, Department of Ophthalmology and Visual Sciences, School of Medicine, Washington University in St. Louis, 660 S. Euclid Ave., Campus Box 8096, St. Louis, MO 63110, USA; willcarter@wustl.edu

**Keywords:** diabetic retinopathy, photoreceptors, lipogenesis

## Abstract

While diabetic retinal disease (DRD) has classically been viewed as a microvascular complication, emerging evidence places the photoreceptor at the center of its pathogenesis. Recognizing this central role provides a critical framework for resolving a major clinical paradox in diabetes: why the retina exhibits profound susceptibility to hyperglycemic damage, whereas closely related neural tissues like the brain are mostly spared. In this review, we synthesize the evidence for photoreceptor-driven DRD pathology by evaluating two primary mechanistic paradigms. In the first, hyperglycemia-induced damage to the blood vessels limits perfusion, creating an ischemic environment that selectively devastates tissues dependent on exceptionally high blood flow and energy delivery—specifically, the photoreceptors. In the second paradigm, hyperglycemia induces a direct shift in the metabolic profile of photoreceptors, triggering oxidative stress and dysregulated lipogenesis that subsequently place pathological strain on the local microvasculature. Regardless of whether the initial insult is vascular or neuronal, the photoreceptor remains the critical node of disease progression. Because current and investigational DRD treatments predominantly target downstream vascular consequences, exploring these dual mechanisms highlights an urgent need and a significant opportunity to develop novel therapies that target the photoreceptor to address DRD at its root.

## 1. Introduction

Diabetic retinal disease (DRD) is classically understood as a disease of the retinal microvasculature due to the characteristic microvascular changes—including microaneurysm formation, selective pericyte loss, and capillary dropout—associated with the condition [1,2]. This unites DRD with other end-organ manifestations of diabetes in which microvascular damage is known to play a central role, such as diabetic nephropathy and neuropathy. However, it remains largely unknown why the retina, peripheral nerves, and kidneys are disproportionately vulnerable to microvascular damage. Furthermore, this selective susceptibility poses a specific paradox within the central nervous system, where the retina suffers profound diabetic pathology while the embryologically identical brain remains largely protected [3,4]. Broadly speaking, this paradox may be explained by two potential mechanistic scenarios.

In the first mechanistic paradigm, the retina’s profound susceptibility to hyperglycemia can be understood through the classical microvascular model of DRD. While systemic hyperglycemia induces widespread damage to the blood vessels and limits perfusion globally, the resulting ischemic environment selectively devastates tissues dependent on exceptionally high blood flow and energy delivery. Consequently, it is the extraordinary adenosine triphosphate (ATP) requirements of the photoreceptors that render the retina uniquely vulnerable to this diabetes-mediated vascular insult.

In the second paradigm, hyperglycemia directly reprograms photoreceptor metabolism to initiate microvascular damage. Because photoreceptor glucose uptake relies on insulin-independent GLUT1 and GLUT3 channels, hyperglycemic conditions result in an unchecked accumulation of cytosolic glucose [5,6]. This massive influx alters cellular metabolism, triggering a cascade of oxidative and lipogenic dysregulation that exponentially increases the demand for oxygen and nutrients, ultimately placing a destructive strain on the surrounding blood vessels.

**Figure 1:**
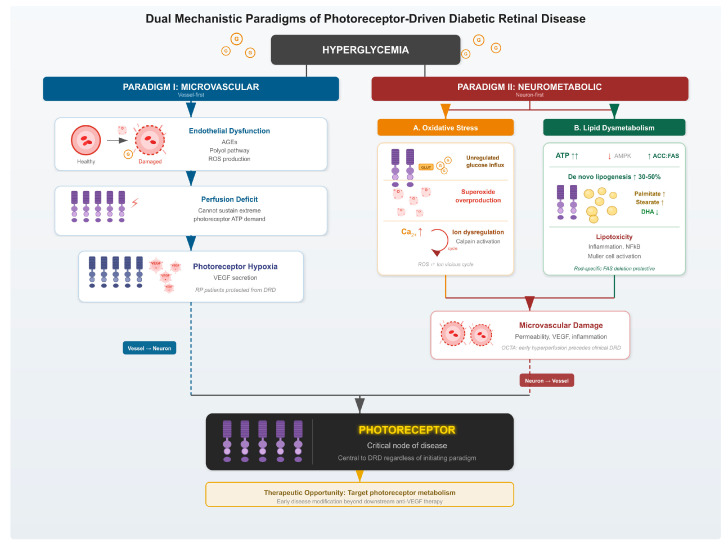
Schematic depicting dual mechanistic scenarios leading to diabetic retinal disease. In the microvascular paradigm, systemic metabolic derangements lead to endothelial dysfunction and consequent hypoxia of the photoreceptors. In the neurometabolic paradigm, hyperglycemia leads to changes in photoreceptor metabolism in the form of increased oxidative stress and/or lipid dysregulation, which damage the photoreceptors and subsequently lead to microvascular changes. AGEs: Advanced Glycation End-Products; ROS: Reactive Oxygen Species; ATP: Adenosine Triphosphate; VEGF: Vascular Endothelial Growth Factor; RP: Retinitis Pigmentosa; DRD: Diabetic Retinal Disease; AMPK: Adenosine Monophosphate-Activated Protein Kinase; ACC: Acetyl-CoA Carboxylase; FAS: Fatty Acid Synthase; DHA: Docosahexaenoic Acid; NF-κB: Nuclear Factor-κB; OCTA: Optical Coherence Tomography Angiography.

This review evaluates the evidence supporting both distinct mechanisms of diabetic retinal injury, which are summarized in Figure 1. By exploring these parallel paradigms, we underscore the crucial role of the photoreceptor in the pathophysiology of DRD.

## 2. Mechanisms Part I: The Microvascular Paradigm

In the classical microvascular model of DRD, systemic hyperglycemia directly impairs endothelial integrity, resulting in a global decrease in oxygen and nutrient delivery alongside diminished waste clearance. Because photoreceptors possess an exceptionally high baseline oxygen demand within a relatively sparse vascular network, the retina is uniquely predisposed to functional deficits following this microvascular compromise. While evidence confirms that diabetes induces ubiquitous capillary damage throughout the body, clinically significant functional deficits remain remarkably localized to tissues that operate at the absolute limits of their bioenergetic capacity [7,8].

### 2.1. The Unique Susceptibility of Retinal Tissue to Decreased Perfusion

Photoreceptors are among the most metabolically active cells in the human body [9], with one recent analysis estimating that a single rod cell expends 3.53 × 10^7^ molecules of ATP per second in average daytime lighting conditions, increasing to 1.09 × 10^8^ ATP/s in the dark [10]. These immense bioenergetic demands arise largely from the photoreceptor’s unique resting state: they remain tonically depolarized in the dark—driven by a continuous influx of sodium and calcium ions known as the “dark current”—and only hyperpolarize in response to light. Because these ions must be continuously cleared against their electrochemical gradients to prevent the depolarization from collapsing, the vast majority of the photoreceptor’s ATP is consumed by active ion transport to sustain this resting potential and drive glutamate release [10].

Furthermore, the unique anatomical organization of the retina exacerbates this vulnerability. To maintain an unobstructed optical axis, evolutionary pressures have minimized the blood vessel network overlying the photoreceptors, resulting in a sparse inner retinal vascular bed [11,12]. The stark convergence of an extraordinary bioenergetic demand forced to rely upon a deliberately restricted vascular supply leaves the retina operating on a precarious metabolic margin, rendering it uniquely intolerant to even minor reductions in perfusion.

### 2.2. Mechanisms of Hyperglycemia-Induced Endothelial Dysfunction

Several biochemical phenomena related to hyperglycemia have been shown to result in endothelial damage. Advanced glycation end-products (AGEs) can directly contribute to diabetic microvascular complications. These products commonly form when excess sugars non-enzymatically react with the amino groups of proteins, nucleic acids, or lipids [13]. Although this Maillard reaction is the predominant mode of AGE formation, several alternative pathways exist. One such pathway involves the conversion of glucose to sorbitol and then to fructose, a highly reactive glycating agent which leads to AGE formation. Other mechanisms include the oxidation of polyunsaturated fatty acids to reactive dicarbonyls (which contribute to both AGE and advanced lipoxidation end-product production) and direct autoxidation of glucose or Schiff bases to create reactive dicarbonyl intermediates [13]. While these reactions occur at low baseline rates in healthy individuals, they accelerate significantly under hyperglycemic conditions, demonstrating a linear correlation between total AGE levels and overall glycemic control [13,14]. When structural proteins of blood vessels such as collagen and elastin react to form AGEs, they become covalently cross-linked with another. These cross-linked proteins promote a wide range of destructive effects, ultimately disrupting vessel structure [15,16], preventing proteolytic degradation [17], promoting inflammation [13], and even providing catalytic sites for reactive oxygen species (ROS) production [18]. AGEs can also bind to AGE receptors (RAGEs) on endothelial cells, further promoting inflammation of the vasculature by activation of mitogen-activated protein kinase (MAPK) and protein kinase C (PKC) [17,19,20].

The aforementioned conversion of excess glucose to sorbitol is catalyzed by the enzyme aldose reductase with reducing power provided by NADPH. Because NADPH is required for the generation of the vasodilator nitric oxide, as well as the antioxidant reduced glutathione, excess flux through sorbitol reductase contributes to vascular dysfunction and increased oxidative stress [20,21]. Excess sorbitol can ultimately deposit in the crystalline lens, resulting in cataract formation, or be oxidized to fructose via sorbitol dehydrogenase, generating the reduced electron carrier NADH and further disturbing oxidative-reductive balance within the cell [20].

A third molecular mechanism accounting for hyperglycemia-induced microvascular damage is a ROS-dependent pathway. Here, increased flux of glucose through both enzymatic and non-enzymatic pathways increases ROS production. In the enzymatic pathway, glucose is broken down via glycolysis, ultimately leading to increased oxidative phosphorylation in the mitochondria and increased ROS production [20]. In the non-enzymatic pathway, auto-oxidation of glucose produces additional ROS [20,22]. The resulting oxidative stress results in increased vascular permeability and leukocyte adhesion, as well as decreased nitric oxide levels [20,23].

Ultimately, these various biochemical mechanisms result in structural and functional damage to the vasculature in the setting of hyperglycemia, with decreased expression of tight junction proteins and increased vascular permeability. This phenomenon has been observed not only in the human and rat retina [24,25], but also in the human umbilical vein [8] and the rat brain [7]. This suggests that diabetic microvascular damage may not be unique to the retina but may be a systemic phenomenon to which the retina is more susceptible given its large metabolic demands.

### 2.3. Does Decreasing Photoreceptor Metabolic Demand Prevent DRD?

If the retina is indeed particularly susceptible to underlying microvascular dysfunction because of its outsized oxygen demand, one would expect decreasing retinal metabolic demand to result in protection from DRD. This was the basis of the theory established by Geoffrey Arden in 1998, who noted that patients with early DRD exhibit changes in dark adaptation and hypothesized that the initial development of DRD is due to the high metabolic demands of rod photoreceptors, particularly in dark-adapted conditions, in the setting of systemic microvascular dysfunction [26]. His eponymous hypothesis predicts that if metabolic demands on photoreceptors were reduced, DRD would improve.

The Arden hypothesis is supported by the observation that patients with both diabetes and retinitis pigmentosa (RP), a set of diseases which result in photoreceptor degeneration, are seemingly protected from developing DRD. This observation was first noted by Sternberg and colleagues in 1984, who surveyed the largest American and European retinal clinics and failed to identify a single patient with both retinitis pigmentosa and proliferative DRD, despite the epidemiology of these diseases suggesting that approximately several dozen patients with both should exist [27]. Driven by these findings, Arden identified 55 patients with RP and co-incident diabetes. Despite 13 of these patients having non-ocular complications of diabetes, strikingly none had been diagnosed with DRD [28]. Notably, these patients were often afflicted by other microvascular complications of diabetes, such as nephropathy and peripheral neuropathy. Moreover, a large Taiwanese insurance database analysis showed that patients with RP and DM showed lower incidence of proliferative DR compared to patients without RP [29].

Ultimately, this hypothesis was tested with the CLEOPATRA study, a phase III randomized controlled trial which was performed at several centers in the United Kingdom. Patients with NPDR and non-central macular edema were randomized to wear either a light-emitting mask (luminance of 75 photopic cd/m^2^ or 186 scotopic cd/m^2^) or a sham mask overnight for two years [30]. The primary outcome was change in macular thickness. The study predicted that, because of the increased energetic demand of the photoreceptor dark current, preventing physiologic night-time dark adaption via the light mask would decrease photoreceptor metabolic demand and reduce macular edema. Ultimately, the CLEOPATRA trial failed to demonstrate that night-time light masks decrease DRD. In CLEOPATRA, no difference was observed in the change in maximum retinal thickness on optical coherence tomogram (OCT) between the light mask and the sham mask groups [30]. While patient non-adherence and high attrition rates undoubtedly confounded the trial’s results (only 19.5% of patients completed the study), it is also plausible that the Arden hypothesis does not completely capture retinal behavior amidst the fluctuating energy demands of hyperglycemia thereby exposing limitations within the microvascular model of DRD pathogenesis.

## 3. Mechanisms Part II: The Neurometabolic Paradigm

Further compounding the mechanistic ambiguities introduced by the negative trial results from CLEOPATRA are preclinical data showing that light adaptation does not improve DRD [31]. Moreover, in early DRD, dark adaptation and visual cycle inhibition were protective against experimental DRD [32]. Liu and colleagues found similar results when inhibiting the visual cycle pharmacologically [33] or genetically [34] rather than by darkness. Collectively, this evidence indicates that disease susceptibility in DRD is not uniformly increased by dark current-induced metabolic demand; instead, it points to a more intricate interplay of varying risk factors across different stages of the disease. Two such factors are photoreceptor-derived oxidative stress and fundamental changes to photoreceptor energy metabolism.

### 3.1. Photoreceptor-Derived Oxidative Stress

The high energy demands of photoreceptors naturally lead to the generation of large quantities of metabolic byproducts, including ROS. Du et al. (2013) found that retinas from diabetic mice produced significantly more superoxide compared to age-matched non-diabetic mice, and that within both groups superoxide production was significantly greater in darkness as opposed to light-adapted conditions [35]. However, the increase in superoxide production associated with diabetes was markedly blunted in retinas with genetic loss of photoreceptors (*Rho*^−/−^) or in mice with chemical ablation of photoreceptors (induced by injection of iodoacetic acid), indicating photoreceptors to be the major source of diabetes-induced superoxide production [35]. These data strongly implicated photoreceptors as the primary source of ROS species that have been shown to play a crucial role in the development of DRD [36,37,38].

The mechanisms behind the diabetes-induced increase in photoreceptor ROS generation remain incompletely understood. However, a growing body of evidence establishes a close link between ROS generation and disruptions to ion flow in the diabetic retina. Berkowitz and colleagues examined uptake of the divalent manganese cation in mouse and rat retina using manganese-enhanced MRI (MeMRI). This contrast agent was chosen as an analog for Ca^2+^ given its chemical similarity, and showed increased uptake in the retina’s metabolically active dark phase compared to in the light, indicating its potential to serve as a marker of retinal ion demand [39,40]. Retinal manganese uptake was decreased in animals with diabetes compared to healthy controls over the first several months of disease before returning to normal levels around 5.5 months after diabetes induction [40,41]. The authors speculated that this return back to normal levels may not indicate adaptive coping to diabetic conditions but instead serves as a sign of dysfunctional ion channel opening which eventually leads to vascular and neuronal cell loss [40]. Oxidative damage appears to play a key role in these changes, as manganese uptake is similar to non-diabetic mice when Cu/Zn superoxide dismutase (SOD1), a key antioxidant, is overexpressed. Together, these observations suggest that ROS drive diabetes-mediated ion dysregulation [40]. Interestingly, Ca^2+^ levels are elevated in photoreceptors of diabetic mice compared to nondiabetic controls and these increases correspond to elevated activity of calpains, a class of Ca^2+^-activated proteases [42]. Increased photoreceptor intracellular calcium levels may explain the decreased ion demand observed in MeMRI. When calpains were pharmacologically inhibited or genetically deleted, diabetes-induced increases in superoxide production, as well as diabetes-induced increases in expression of the pro-inflammatory factors inducible nitric oxide synthase and ICAM-1, disappeared [42]. These findings suggest that diabetes-induced ion dysregulation may play a role in increasing ROS production. Given the evidence for both ROS-induced ion dysregulation and ion dysregulation-induced ROS production, a vicious cycle may exist between the two processes, although questions remain regarding which comes first.

### 3.2. Dysregulated Photoreceptor Lipid Metabolism

Beyond metabolic demand or ROS production, DRD pathogenesis may also be driven by hyperglycemia-induced biosynthetic activity in photoreceptors, resulting in lipid toxicity. As early as 1969, Futterman et al. provided evidence for this metabolic shift, reporting an accumulation of shorter-chain fatty acids—specifically palmitate (16:0) and stearate (18:0)—at the expense of long- and very-long-chain fatty acids [43]. Following the replication of these results by several groups [44,45,46], Tikhonenko et al. (2009) further demonstrated that the downregulation of retinal elongases likely drives the accumulation of shorter-chain fatty acids in the diabetic retina [45]. Given the anti-inflammatory effects of docosahexaenoic acid (DHA_22:6n3_), decreases in this very long chain fatty acid may contribute to the increased inflammatory signaling in DRD that ultimately results in tissue damage [45,47]. Evidence from cultured human Müller cells links lipid dysregulation to the early Müller cell activation seen in DRD, with palmitic acid—particularly when administered with glucose—stimulating expression of genes related to NFκB signaling and inflammation, angiogenesis, and MAPK signaling [48]. Conversely, Müller cell inflammation is inhibited by the administration of certain epoxygenated fatty acids [49].

A central pillar of our group’s DRD research is the pathological acceleration of retinal *de novo* lipid biosynthesis in diabetes. Our laboratory has demonstrated that retinal lipogenesis is markedly upregulated—increasing by 30–50%—across three distinct models of diabetes: streptozotocin-induced, leptin receptor deficiency, and high-fat diet-fed mice. This metabolic surge is driven specifically by hyperglycemia, where excess glucose provides the substrates and signaling cues that inactivate adenosine monophosphate-activated protein kinase (AMPK) and constitutively activate acetyl-CoA carboxylase (ACC) and fatty acid synthase (FAS), the rate-limiting enzymes of the pathway [44,50].

Crucially, this biosynthetic shift is not merely a marker of diabetes but a primary driver of neurovascular injury localized to the outer retina. By utilizing a rod-specific partial deletion of FAS (*Fasn^fl/+^*; *Rho*-iCre), we established that reduction in fatty acid biosynthesis specifically within photoreceptors significantly reduces the severity of experimental DRD, resulting in a measurable reduction in microvascular permeability and inflammatory markers. These findings suggest that the accumulation of toxic lipid species—the direct result of unchecked photoreceptor anabolism—is a fundamental pathogenic event [44].

Furthermore, the protective effects of visual cycle inhibition and dark adaptation appear to be mediated through the correction of this lipid dysregulation. In the diabetic state, the high ATP charge—or increased ATP:ADP ratio—of the photoreceptor fuels these harmful anabolic processes. Creating a metabolic outlet for this excess energy, either through the metabolic demand of prolonged dark adaptation or by pharmacologically inhibiting the visual cycle with retinylamine, reverses the overactivation of the lipogenic pathway. By restoring metabolic homeostasis and reducing the biosynthetic burden, these interventions effectively mitigate DRD progression, identifying photoreceptor lipid metabolism as a highly viable therapeutic target [32,50].

## 4. Mechanisms Part III: Clinical Correlates

### 4.1. Imaging Evidence for the Photoreceptor Origins of DRD

In the model where clinical DRD pathology is thought to originate strictly within the blood vessels, one would expect retinal blood flow to be consistently decreased from the earliest stages of diabetes. However, evidence from optical coherence tomography angiography (OCTA) has increasingly demonstrated the opposite. Rosen et al. (2019) identified an initial increase in perfused capillary density in diabetic patients without DRD compared to non-diabetic controls—a density that only falls below control levels once clinical DRD has actually developed [51]. Zhang et al. (2020) reported that patients with mild NPDR showed reduced vessel density in the superficial capillary plexus, whereas individuals with diabetes but no clinically visible DR displayed a paradoxical increase in vessel density in this layer [52]. In contrast, perfusion defects in the deep capillary plexus, which lies closest to the highly metabolic outer retina, appear earlier than defects in the superficial plexus. Together, these findings suggest that early photoreceptor metabolic dysfunction may precede, and possibly contribute to, subsequent microvascular compromise [53]. In addition to perfusion defects in the retinal circulation, studies utilizing OCTA and indocyanine green angiography have also observed decreased choroidal perfusion in diabetic retinal disease [54,55]. However, in the earliest stages of DRD, these choroidal defects were quite limited compared to those in the superficial and deep capillary plexuses, pointing to the retinal circulation as the earliest vascular insult [55].

### 4.2. A Potential Photoreceptor-Based Protective Factor: Retinol Binding Protein 3

Clinical evidence further implicates the photoreceptor in DRD pathophysiology—specifically through studies identifying a clear correlation between disease severity and levels of retinol-binding protein 3 (RBP3). Also known as interphotoreceptor retinoid-binding protein (IRBP), this protein serves as a critical marker of outer retinal health. RBP3 is a glycoprotein secreted by photoreceptors into the interphotoreceptor matrix where it mediates the transport of cis and trans retinol between the photoreceptors and the RPE [56,57,58,59]. RBP3 was first identified as a potential factor in DRD pathogenesis through a comparative proteomics analysis of vitreous fluid—where it emerged as one of only three proteins significantly reduced in patients with proliferative diabetic retinopathy (PDR) compared to healthy controls [60]. Subsequent investigations by the same group confirmed that vitreous RBP3 levels correlate closely with DRD severity. Crucially, both RBP3 mRNA and protein expression are diminished in post-mortem retinal samples from diabetic patients—even in those without clinical signs of DRD—which indicates that RBP3 depletion occurs at the very earliest stages of the disease [61].

The Joslin 50-Year Medalist Study further clarified the role of RBP3 in DRD by analyzing patients living with type 1 diabetes for over half a century. Despite decades of hyperglycemia, 35% of these “Medalists” developed no or only minimal DRD—contrasting sharply with the 50% who progressed to proliferative disease [62]. This bimodal distribution led Yokomizo et al. to hypothesize that a specific endogenous factor protects some patients from severe DRD.

Analyzing post-mortem ocular tissue and vitreous samples, the investigators found that RBP3 levels directly reflect disease resistance: concentrations were highest in non-diabetic controls, intermediate in those with mild NPDR, and lowest in those with proliferative DRD [62]. Notably, these protective RBP3 levels did not correlate with A1c, suggesting a mechanism independent of glycemic control.

Mechanistically, intravitreal injection of recombinant RBP3 in rats suppressed retinal VEGF-A expression and blocked the vascular permeability typically induced by VEGF. RBP3 achieves this by inhibiting VEGF receptor tyrosine phosphorylation and reducing glucose uptake in both Müller cells and retinal endothelial cells [62]. Subsequent clinical data further link RBP3 to structural and inflammatory health—showing that higher vitreous RBP3 levels correlate with increased photoreceptor segment thickness and a reduction in pro-inflammatory cytokines such as interleukin-12 (IL-12), tumor necrosis factor-α (TNF-α), and TNF-β [63]. In addition, decreased levels of RBP3 have been hypothesized to be the cause of the dysregulation of subretinal space hydration that has been observed in diabetes [64,65]. Collectively, these findings establish this photoreceptor-secreted glycoprotein as a potent antagonist of DRD progression.

### 4.3. A Potential Mechanism for the Therapeutic Effects of Focal Laser

Although the precise therapeutic basis of macular photocoagulation remains a subject of ongoing debate, its clinical efficacy in treating diabetic macular edema is well established. Early models, which heavily influenced the protocol of the landmark Early Treatment Diabetic Retinopathy Study, posited that the laser’s primary function was the direct coagulation and closure of leaking microaneurysms [66,67]. However, modern high-resolution imaging challenges this vasculature-centered assumption. OCT frequently reveals that laser-induced defects are localized strictly to the outer retina, often limited to the retinal pigment epithelium and photoreceptor outer segments—sparing the inner retinal layers where the primary vascular plexuses reside [67]. This anatomical discrepancy suggests a shift in perspective wherein the clinical benefits of focal laser may stem not from the repair of leaky vessels, but from the strategic ablation of the underlying diseased photoreceptors.

By reducing the population of these high-metabolic-demand cells, focal laser treatment may fundamentally alter the retinal environment, decreasing overall oxygen demand and suppressing the localized secretion of VEGF [67]. This localized metabolic silencing offers a compelling explanation for the subsequent stabilization of the microvasculature, even when the vessels themselves are not the direct targets of the laser.

## 5. Conclusions

It is increasingly evident that the functional integrity of both the retinal microvasculature and the underlying metabolic landscape is fundamentally disrupted in the diabetic environment. Yet, a persistent “chicken-and-egg” conundrum remains—which of these systems initially falters to trigger the cascade of DRD? While the definitive sequence of events continues to be a subject of intense investigation, the current therapeutic paradigm remains strikingly one-sided. All standard treatments—and even those in the advanced pipeline, such as tyrosine kinase inhibitors, Wnt-pathway agonists, and combinatorial agents—focus almost exclusively on the vascular consequences of the disease.

If vascular compromise is merely a downstream effect of primary dysfunction within the high-metabolic-demand cells of the outer retina, then focusing solely on the vessels targets only the symptomatic endgame. This approach likely misses the critical window for early intervention. Given the mounting evidence—from the paradoxical early hyperperfusion seen on OCTA to the protective role of RBP3 and the pathogenic surge in *de novo* lipogenesis—the metabolic health of the photoreceptor demands a more central role in our therapeutic strategy.

Addressing these early metabolic abnormalities—perhaps by modulating the high ATP charge or curbing toxic lipid biosynthesis—offers more than just a potential adjunct to anti-VEGF therapy. Such strategies represent a shift toward true disease modification, potentially providing a powerful prophylactic intervention that stabilizes the retina long before irreversible vascular damage occurs.

## Data Availability

No new data were created or analyzed in this study. Data sharing is not applicable to this article.

## Abbreviations

The following abbreviations are used in this manuscript:

ACC	Acetyl-CoA Carboxylase
ADP	Adenosine Diphosphate
AGEs	Advanced Glycation End-Products
AMPK	Adenosine Monophosphate-Activated Protein Kinase
ATP	Adenosine Triphosphate
DHA	Docosahexaenoic Acid
DRD	Diabetic Retinal Disease
FAS	Fatty Acid Synthase
ICAM-1	Intercellular Adhesion Molecule 1
IL-12	Interleukin-12
IRBP	Interphotoreceptor Retinoid-Binding Protein
MAPK	Mitogen-Activated Protein Kinase
MeMRI	Manganese-enhanced MRI
mRNA	Messenger Ribonucleic Acid
NF-κB	Nuclear Factor-κB
OCT	Optical Coherence Tomogram
OCTA	Optical Coherence Tomography Angiography
PKC	Protein Kinase C
RAGEs	Receptors for Advanced Glycation End-Products
RBP3	Retinol Binding Protein 3
ROS	Reactive Oxygen Species
SOD1	Cu/Zn Superoxide Dysmutase
TNF-α	Tumor Necrosis Factor-α
TNF-β	Tumor Necrosis Factor-β
VEGF	Vascular Endothelial Growth Factor
